# Post Weight Loss Body Contouring Surgery: Complication Rates Following Bariatric Surgery, Injectable Glucagon-like Peptide-1 Receptor Agonist Pharmacotherapy, Combination Therapy, and Lifestyle Modification

**DOI:** 10.1093/asj/sjag049

**Published:** 2026-02-25

**Authors:** Erin N Abbott, Emmanuel Giannas, Nomongo Dorjsuren, Daniella King, Ruoying Li, Adrienne N Christopher, Franklin R Gergoudis, Allen Gabriel, Galen Perdikis, Patrick E Assi

## Abstract

**Background:**

Because the demand for body-contouring surgery has increased following the widespread adoption of glucagon-like peptide-1 receptor agonists, the impact of different weight-loss methods on patient selection, operative techniques, and postoperative outcomes remains unclear.

**Objectives:**

This study compared complication rates across weight-loss modalities and identified predictors of adverse outcomes in body-contouring patients.

**Methods:**

A single-center, retrospective, cohort study of patients who underwent post-weight-loss body-contouring surgery between January 2019 and December 2024 was performed. Eligible patients were adults who achieved weight loss and subsequently underwent panniculectomy, brachioplasty, thighplasty, or breast surgery. Patients were classified into 4 groups based on weight-loss modality: surgical, injectable GLP-1 pharmacotherapy, combination, or lifestyle. The primary outcome was the incidence of postoperative complications within 90 days for each procedure.

**Results:**

Among 1002 post-weight-loss patients undergoing body contouring, weight-loss methods included surgery (67.9%), lifestyle (14.3%), GLP-1 pharmacotherapy (7.8%), and combination therapy (10.1%). Baseline characteristics differed significantly across groups. Across all procedures, complication rates did not differ by weight-loss modality. Panniculectomy, brachioplasty, thighplasty, and breast procedures demonstrated expected procedure-specific complication patterns, with higher BMI at the time of surgery and diabetes independently predicting increased risk.

**Conclusions:**

Weight-loss modality does not appear to impact the incidence of postoperative complications following body-contouring surgery. BMI at the time of surgery and diabetes are independent predictors of adverse outcomes.

**Level of Evidence:**

3 (Therapeutic) 
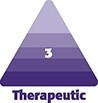

The rapid adoption of pharmacological weight-loss agents, such as injectable glucagon-like peptide-1 receptor agonists (GLP-1 RAs), has resulted in an increased demand for body-contouring surgery. However, the substantial weight reduction associated with injectable GLP-1 RA therapy has raised concerns about its effect on tissue quality, nutritional status, and wound healing.^1,2^ Recent evidence suggests that weight stabilization tends to occur earlier in injectable GLP-1 RA−treated patients compared with those undergoing bariatric surgery.^3^ However, preliminary data indicates that this cohort is at a particularly high risk of weight regain following medication discontinuation.^3^ Despite the growing number of postinjectable GLP-1 RA weight-loss patients seeking body contouring, their clinical profile and postoperative risk remain poorly characterized.

Previous research has primarily focused on outcomes in patients who have achieved weight loss following bariatric surgery or lifestyle modifications.^4^ Among patients who have undergone body-contouring postbariatric surgery, complication rates of ∼30% have been reported.^4^ With the widespread utilization of injectable GLP-1, it is unclear whether medically induced rapid weight loss confers similar or distinct risks. Although injectable GLP-1 may improve glycemic control in diabetic patients, rapid weight loss may result in nutritional deficiencies or impaired wound healing.^5^

This study aimed to determine whether the weight-loss modality of patients undergoing body-contouring surgery impacts the incidence of postoperative complications. Secondary objectives were to identify independent predictors of postoperative complications and to describe the characteristics of weight-loss patients seeking body contouring at a single institution.

## METHODS

A retrospective review was conducted of all patients who underwent post-weight-loss body contouring at a single academic center between January 2019 and December 2024. Ethical approval was granted by the Vanderbilt University Medical Center (Nashville, TN) Institutional Review Board. Eligible patients were adults who achieved weight loss and subsequently underwent panniculectomy, brachioplasty, thighplasty, or breast surgery, including mastopexy, reduction, or augmentation. Patients with incomplete records or inadequate follow-up were excluded. Weight-loss methods were classified into 4 groups: surgical, injectable GLP-1 RA pharmacotherapy, combination (defined as surgical and pharmacological), or lifestyle. Minimum exposure for pharmacological intervention was defined as documented injectable GLP-1 RA use in clinic notes in the electronic medical record. Massive weight loss (MWL) was defined as 50% of excess weight relative to an ideal BMI of 25 kg/m^2^ or >100 lb of total weight loss.^6,7^ Per institutional guidelines, all patients were at a stable weight for at least 6 months prior to body-contouring surgery. Injectable GLP-1 RA pharmacological weight-loss therapy was withheld for at least 7 days prior to surgery in accordance with safety standards and institutional protocols.

Demographic and perioperative variables included age, sex, BMI at surgery, maximum BMI, total percent weight loss, and comorbidities, including diabetes, hypertension, smoking, and iron-deficiency anemia. Procedure-specific variables included incision pattern, concurrent liposuction, or the presence of an implant for breast procedures.

The primary outcome was the rate of postoperative complications, defined as occurrence of surgical-site infection (SSI), wound dehiscence, skin necrosis, seroma, hematoma, or return to the operating room within 90 days for each procedure.

Descriptive statistics were presented as mean ± standard deviation. Group differences were compared using analysis of variance for continuous and χ^2^ tests or Fisher's exact tests when <5 events for categorical variables. Pairwise comparisons between weight-loss methods were adjusted for multiple tests using the Holm correction. For each procedure, multivariable logistic regression models were constructed to identify independent predictors of postoperative complications. For outcomes with low event counts, Firth's penalized likelihood of logistic regression was used. All analyses were performed using R version 4.3.2 (Foundation for Statistical Computing, Vienna, Austria), with significance defined as *P* < .05.

## RESULTS

### Cohort Characteristics

A total of 1002 patients were included in the analysis. Mean age at surgery was 47.7 ± 11.2 years (range, 18-79 years). The majority of the cohort was female (*n* = 929, 92.7%) vs male (*n* = 73, 7.3%). The minimum follow-up time was 90 days. Mean BMI at the time of body contouring was 31.6 ± 6.7 kg/m^2^, and the mean maximum BMI prior to weight loss was 51.4 ± 11.3 kg/m^2^. Average percent of total body weight loss was 37.2 ± 11.4%, and 91.1% of patients met the criteria for MWL. A total of 256 patients (25.5%) underwent multiple contouring procedures on the same operative date (Table 1).

**Table 1.** Baseline Characteristics of the Body-Contouring Cohort

	Overall (*n* = 1002)
Method, *n* (%)	
Surgical	680 (67.9)
Combination	101 (10.1)
Lifestyle	143 (14.3)
Medication	78 (7.8)
Sex at birth, *n* (%)	
Female	929 (92.7)
Male	73 (7.3)
Age at body-contouring surgery (years, mean (SD))	47.7 (11.2)
Massive weight loss, *n* (%)	
No	89 (8.9)
Yes	913 (91.1)
Maximum BMI (kg/m^2^, mean (SD))	51.4 (11.3)
BMI at body-contouring surgery, *n* (%)	31.6 (6.7)
Change in weight (%, mean (SD))	37.2 (11.4)
Smoker, *n* (%)	
No	959 (95.7)
Yes	43 (4.3)
Diabetes, *n* (%)	
No	735 (73.4)
Yes	265 (26.4)
Hypertension, *n* (%)	
No	551 (55.0)
Yes	449 (44.8)
Iron-deficient anemia, *n* (%)	
No	774 (77.2)
Yes	221 (22.1)
Concurrent body contouring, *n* (%)	
No	746 (74.5)
Yes	256 (25.5)

SD, standard deviation.

### Weight-Loss Method Distribution

Most patients achieved weight loss after bariatric surgery (*n* = 680, 67.9%), including 430 gastric bypass (63.2%) and 249 sleeve gastrectomy (36.6%) patients, with 1 lap banding and 1 duodenal switch. Injectable GLP-1 RA–based pharmacotherapy was used in 78 patients (7.8%). Combination therapy accounted for 101 patients (10.1%). Lifestyle modification alone accounted for 143 patients (14.3%). For patients using injectable GLP-1 RA receptor agonists in either the pharmacotherapy or combination group, semaglutide was the most frequently used (67.6%), followed by tirzepatide (25.8%), dulaglutide (4.4%), and liraglutide (2.2%).

### Baseline Differences Between Weight-Loss Methods

Significant baseline characteristics differences were observed across weight-loss strategies (Table 2). Age at surgery varied between groups (*P* = .045), with patients in the surgical cohort younger than the combination group on post hoc analysis (*P* = .029). Sex at birth was significantly different between groups (*P* < .001) with a higher proportion of male patients in the lifestyle group compared with the surgical and GLP groups (*P* < .001 and *P* = .009, respectively).

**Table 2.** Baseline Characteristics by Weight-Loss Method

	Surgical (*n* = 680)	Combination (*n* = 101)	Lifestyle (*n* = 143)	Medication (*n* = 78)	*P*-value
Sex at birth, *n* (%)					<.001
Female	642 (94.4)	94 (93.1)	117 (81.8)	76 (97.4)	
Male	38 (5.6)	7 (6.9)	26 (18.2)	2 (2.6)	
Age at body-contouring surgery (years, mean (SD))	47.2 (10.9)	50.5 (9.9)	47.8 (12.7)	48.4 (11.8)	.045
Massive weight loss , *n* (%)					<.001
No	31 (4.6)	5 (5.0)	36 (25.2)	17 (21.8)	
Yes	649 (95.4)	96 (95.0)	107 (74.8)	61 (78.2)	
Maximum BMI (kg/m^2^, mean (SD))	52.5 (11.0)	52.5 (11.1)	48.5 (12.8)	45.4 (8.5)	<.001
BMI at body-contouring surgery, *n* (%)	31.4 (6.4)	31.1 (6.1)	33.7 (8.3)	31.1 (6.1)	.001
Percent change in weight (%, mean (SD))	39.3 (9.9)	39.8 (10.3)	29.0 (13.0)	30.3 (12.4)	<.001
Smoker, *n* (%)					
No	655 (96.3)	98 (97.0)	131 (91.6)	75 (96.2)	.105
Yes	25 (3.7)	3 (3.0)	12 (8.4)	3 (3.8)	
Diabetes, *n* (%)					
No	535 (78.7)	56 (55.4)	102 (71.3)	42 (53.8)	<.001
Yes	145 (21.3)	44 (43.6)	41 (28.7)	35 (44.9)	
Hypertension, *n* (%)					
No	395 (58.1)	40 (39.6)	74 (51.7)	42 (53.8)	.004
Yes	283 (41.6)	61 (60.4)	69 (48.3)	36 (46.2)	
Iron-deficient anemia, *n* (%)					
No	512 (75.3)	78 (77.2)	118 (82.5)	66 (84.6)	.045
Yes	165 (24.3)	23 (22.8)	22 (15.4)	11 (14.1)	
Concurrent body contouring, *n* (%)					
No	491 (72.2)	69 (68.3)	125 (87.4)	61 (78.2)	<.001
Yes	189 (27.8)	32 (31.7)	18 (12.6)	17 (21.8)	

SD, standard deviation.

BMI differed significantly by weight-loss method for maximum BMI and BMI at the time of body contouring (*P* < .001 and *P* = .001, respectively). Patients in the medication group had a substantially lower maximum BMI than those in the combination (−7 kg/m^2^, *P* < .001) and surgical (−7 kg/m^2^, *P* < .001) groups. Similarly, lifestyle patients had a lower maximum BMI than both the combination (−4 kg/m^2^, *P* = .028) and surgical (−4 kg/m^2^, *P* < .001) cohorts. No significant difference was seen between surgical and combination groups or medication and lifestyle groups.

At the time of body contouring, BMI also differed across methods (*P* = .001). Lifestyle patients had a higher BMI than medication (+2.5 kg/m^2^, *P* = .036), surgery (+2.3, *P* < .001), and combination (+2.6 kg/m^2^, *P* = .015) patients. The magnitude of percent weight lost varied significantly (*P* < .001). Combination and surgical patients achieved the greatest reduction in body weight. Combination patients lost 9.5% more body weight than medication patients (*P* < .001) and 10.8% more than lifestyle patients (*P* < .001). Similarly, surgical patients lost 9.0% more than medication patients (*P* < .001) and 19.3% more than lifestyle patients (*P* < .001).

Consistent with these findings, the proportion of patients meeting the criteria for MWL significantly differed across groups (*P* < .001). MWL rates were significantly higher in the combination and surgical cohorts than in the medication (9.5%, *P* < .001 and 9.0%, *P* < .001, respectively) or lifestyle groups (10.8%, *P* < .001 and 10.3%, *P* < .001, respectively).

In terms of comorbidities, diabetes was less common among surgical patients than combination or GLP-1 RA patients (both *P* < .001). Hypertension was more prevalent in the combination group than the surgical group (*P* = .004). Iron-deficient anemia showed an overall difference between groups (*P* = .045), but no pairwise comparisons were significant. Rates of concurrent body-contouring procedures differed across methods (*P* < .001), with combination and surgical patients more likely to undergo simultaneous contouring procedures compared with lifestyle patients (*P* = .025 and *P* = .013, respectively).

### Procedure-Specific Outcomes

#### Panniculectomy

A total of 894 panniculectomies were analyzed. Baseline trends mirrored the overall cohort, with differences in characteristics by weight-loss method. There were significant differences in sex (*P* < .001), age (*P* = .007), maximum BMI (*P* < .001), BMI at surgery (*P* < .001), diabetes (*P* < .001), hypertension (*P* = .011), and concurrent body-contouring procedures (*P* = .003). Concurrent abdominal or truncal liposuction was performed in 12.8% of cases and did not differ by weight-loss method. Incision types included horizontal (69.7%), Fleur-de-Lis (29.0%), and circumferential or belt lipectomy (1.3%), with significant differences between weight-loss method groups (*P* < .001). Fleur-de-Lis incisions were more common in surgical (*P* < .001) and combination weight loss (*P* = .005; Table 3).

**Table 3.** Baseline Trends for Panniculectomy Patients by Weight-Loss Method

	Surgical (n = 605)	Combination (n = 89)	Lifestyle (n = 126)	Medication (n = 73)	*P*-value
Sex at birth, *n* (%)					
Female	574 (94.9)	85 (94.4)	102 (81.0)	71 (97.3)	<.001
Male	31 (5.1)	5 (5.6)	24 (19.0)	2 (2.7)	
Age at body-contouring surgery (years, mean (SD))	47.1 (10.8)	51.3 (9.62)	47.3 (12.6)	49.0 (11.9)	.007
Massive weight loss, *n* (%)					
No	29 (4.8)	5 (5.6)	31 (24.6)	15 (20.5)	<.001
Yes	576 (95.2)	85 (94.4)	95 (75.4)	58 (79.5)	
Maximum BMI (kg/m^2^, mean (SD))	51.8 (10.5)	52.0 (10.5)	48.7 (12.1)	45.5 (8.74)	<.001
BMI at body-contouring surgery, *n* (%)	31.0 (5.88)	31.0 (5.57)	33.7 (7.64)	31.2 (6.13)	<.001
Percent change in weight (%, mean (SD))	39.1 (9.88)	39.3 (10.4)	29.1 (13.1)	30.4 (12.5)	<.001
Smoker, *n* (%)					
No	581 (96.0)	87 (96.7)	115 (91.3)	70 (95.9)	.157
Yes	24 (4.0)	3 (3.3)	11 (8.7)	3 (4.1)	
Diabetes, *n* (%)					
No	475 (78.5)	50 (55.6)	89 (70.6)	39 (53.4)	<.001
Yes	130 (21.5)	39 (43.3)	37 (29.4)	33 (45.2)	
Hypertension, *n* (%)					
No	358 (59.2)	38 (42.2)	65 (51.6)	38 (52.1)	.011
Yes	245 (40.5)	52 (57.8)	61 (48.4)	35 (47.9)	
Iron-deficient anemia, *n* (%)					
No	462 (76.4)	71 (78.9)	104 (82.5)	63 (86.3)	.065
Yes	140 (23.1)	19 (21.1)	19 (15.1)	9 (12.3)	
Concurrent body contouring, *n* (%)					
No	428 (70.7)	60 (66.7)	108 (85.7)	56 (76.7)	.003
Yes	177 (29.3)	30 (33.3)	18 (14.3)	17 (23.3)	

SD, standard deviation.

The rates of postoperative complications were 17.4%, including SSI at 5.8%, wound dehiscence at 8.4%, seroma at 5.1%, and hematoma at 4.6% (Table 4). No differences in complication rates were observed across weight-loss groups. In terms of independent predictors of any complications, BMI at surgery was a significant predictor with a 6% increase in odds with a 1 kg/m^2^ increase in BMI (odds ratio [OR] 1.06, *P* = .002). Diabetes increased the odds by 62% (OR 1.62, *P* = .022). Weight-loss mechanism, incision type, and concurrent procedures were not associated with complications (Table 5).

**Table 4.** Complication Rates for Each Body-Contouring Procedure

	A. Panniculectomy (*n* = 894) *n* (%)	B. Brachioplasty (*n* = 109) *n* (%)	C. Thighplasty (*n* = 83) *n* (%)	D. Breast (*n* = 179) *n* (%)
Overall complications	156 (17.4)	13 (11.9)	30 (36.1)	33 (18.4)
Surgical-site infection	52 (5.8)	5 (4.6)	10 (12.0)	10 (5.6)
Wound dehiscence	75 (8.4)	6 (5.5)	21 (25.3)	15 (8.4)
Skin necrosis	23 (2.6)	0	1 (1.2)	5 (2.8)
Seroma	37 (4.1)	2 (1.8)	7 (8.4)	7 (3.9)
Hematoma	41 (4.6)	1 (0.9)	3 (3.6)	8 (4.5)
Return to OR	49 (5.5)	0	2 (2.4)	6 (3.4)

OR, Operating Room.

**Table 5.** Baseline Characteristics of Panniculectomy Patients With and Without Postoperative Complications

	No (*n* = 738)	Yes (*n* = 156)	*P*-value
Sex at birth, *n* (%)			
Female	687 (93.1)	145 (92.9)	.997
Male	51 (6.9)	11 (7.1)	
Age at body-contouring surgery (years, mean (SD))	47.7 (11.2)	47.7 (10.8)	1
Massive weight loss, *n* (%)			
No	63 (8.5)	17 (10.9)	.433
Yes	675 (91.5)	139 (89.1)	
Maximum BMI (kg/m^2^, mean (SD))	50.1 (10.1)	54.7 (12.8)	<.001
BMI at body-contouring surgery, *n* (%)	30.9 (5.65)	33.8 (7.97)	<.001
Percent change in weight (%, mean (SD))	37.0 (11.2)	37.1 (11.9)	.913
Method, *n* (%)			
Surgical	497 (67.3)	108 (69.2)	.810
Combination	73 (9.9)	17 (10.9)	
Lifestyle	105 (14.2)	21 (13.5)	
Medication	63 (8.5)	10 (6.4)	
Smoker, *n* (%)			
No	708 (95.9)	145 (92.9)	.159
Yes	30 (4.1)	11 (7.1)	
Diabetes, *n* (%)			
No	554 (75.1)	99 (63.5)	.003
Yes	182 (24.7)	57 (36.5)	
Hypertension, *n* (%)			
No	421 (57.0)	78 (50.0)	.120
Yes	315 (42.7)	78 (50.0)	
Iron-deficient anemia, *n* (%)			
No	587 (79.5)	113 (72.4)	.159
Yes	148 (20.1)	39 (25.0)	
Concurrent body contouring, *n* (%)			
No	527 (71.4)	125 (80.1)	.033
Yes	211 (28.6)	31 (19.9)	
Concurrent abdominal liposuction, *n* (%)			
No	647 (87.7)	133 (85.3)	.491
Yes	91 (12.3)	23 (14.7)	
Incision type, *n* (%)			
Circumferential	9 (1.2)	3 (1.9)	.001
Horizontal	533 (72.2)	90 (57.7)	
Fleur-de-Lis	196 (26.6)	63 (40.4)	

SD, standard deviation.

By specific complication, the odds of an SSI increased by 9% for every 1 kg/m^2^ increase in BMI (OR 0.109, *P* < .001), and diabetes increased the odds by 169% (OR 2.69, *P* = .003). Concurrent body contouring was protective (OR 0.31, *P* = .017). The odds of wound dehiscence increased by 6% for every 1 kg/m^2^ increase in BMI (OR 1.06, *P* = .0066) and with concurrent liposuction (OR 1.91, *P* = .044), but decreased in male patients (OR 0.24, *P* = .041). Hypertension increased the odds of seroma by over 2-fold (OR 2.14, *P* = .045).

#### Brachioplasty

A total of 109 patients underwent brachioplasty, with 45% liposuction assisted and 55% standard excision. Diabetes prevalence differed by weight-loss method (*P* < .001) but technique distribution was not significantly different. The rate of any complication was 11.9%. SSIs occurred in 4.6% and wound dehiscence in 5.5% of procedures (Table 4). No characteristics or surgical factors were significantly different between those with and without complications (Supplemental Table 1, available online at https://doi.org/10.1093/asj/sjag049).

#### Thighplasty

For thighplasty, 83 patients were included. Incision types were vertical (68.7%), horizontal (16.9%), and combined (14.5%). Concurrent thigh liposuction occurred in 36.1% of procedures. The rate of any complication was 36.1%, the majority of which was wound dehiscence occurring in 25.3% of the group. SSIs and seromas occurred in 12.0% and 8.4% of surgeries (Table 4). Complication rates did not differ by weight-loss method (Supplemental Table 2, available online at https://doi.org/10.1093/asj/sjag049).

Higher BMI at surgery was associated with an increased risk of any complication (31.1 vs 37.1 kg/m^2^, *P* = .009). In multivariable Firth's logistic regression, higher maximum BMI trended toward increased odds (OR 1.14, *P* = .078), and a greater percent weight loss trended toward lower odds (OR 0.83, *P* = .055). For wound dehiscence specifically, greater percent weight loss was significantly protective (OR 0.80, *P* = .030).

#### Breast Procedures

A total of 179 breast procedures were analyzed, including 90 mastopexies, 53 reductions, and 31 augmentations or augmentation mastopexies. Procedure type and implant presence were evenly distributed across methods (*P* > .8). Concurrent body contouring occurred less often in surgical patients (93%) than in nonsurgical groups (65%-83%, *P* = .001). The overall complication rate was 18.4%, with SSIs in 5.6% and wound dehiscence in 8.4% of the population (Table 4). No significant differences were found between weight-loss methods or those with and without complications. In particular, implant-based and nonimplant cases had similar complication rates (Supplemental Table 3, available online at https://doi.org/10.1093/asj/sjag049).

## DISCUSSION

This study demonstrated that in weight-loss patients undergoing body-contouring surgery, complication rates did not differ by the weight-loss method, whether achieved through bariatric surgery, injectable GLP-1 RA pharmacotherapy, lifestyle modifications, or a combination of the above. Instead, BMI at the time of body-contouring surgery and diabetes mellitus emerged as independent predictors of adverse outcomes across procedures. Most of the patients at this institution undergoing body-contouring surgery achieved weight reduction following bariatric surgery, and rates of MWL were highest in the surgical and combination cohorts. This is the first study to directly compare complication profiles among patients undergoing body contouring after weight loss achieved through diverse modalities, including injectable GLP-1 RA−based weight loss, reflecting the evolving landscape of contemporary weight management practices.

Complication rates following body-contouring surgery were not significantly impacted by the weight-loss modality employed. Recent studies evaluating injectable GLP-1 RA−associated weight loss have reported similar outcomes.^8-10^ For example, a single-center, retrospective cohort study comparing injectable GLP-1 RA and non-GLP-1 RA users found no difference in postoperative complications. However, details on the weight-loss modalities for the non-GLP-1 RA cohort were not provided, limiting the clinical interpretability of their findings. By directly comparing surgical, injectable GLP-1 RA pharmacotherapy, lifestyle, and combination approaches, this study provides a more comprehensive assessment of how weight-loss modality relates to postoperative risk. This information may allow surgeons to counsel patients appropriately and set expectations regarding the risk of adverse outcomes following weight loss with injectable GLP-1 RA pharmacotherapy.

BMI at the time of surgery and diabetes were the only independent predictors of postoperative adverse events. Diabetes is a recognized risk factor for the development of adverse outcomes following surgery, such as SSI.^11^ Chronic hyperglycemia results in macro- and microangiopathic complications, therefore reducing tissue perfusion and nutrients delivered to the wound bed.^12,13^ Obesity is also widely recognized in surgical literature as a risk factor for the development of complications.^14^ This is thought to be because of the pro-inflammatory state associated with obesity.^15^ As a result, this may suppress the immune system during the inflammatory phase of wound healing.^15^ This study corroborates previous findings on the effect of BMI and diabetes in surgical wound healing. Furthermore, they suggest that perioperative optimization of BMI and glycemic control is of utmost importance in patients seeking body-contouring surgery regardless of weight-loss modality.

Thighplasty had the highest complication rate amongst the body-contouring procedures examined, at 36.1%. Similar findings were reported by Garoosi et al who leveraged a database and examined complication rates in 243,886 patients.^16^Most of the complications were because of wound dehiscence that occurred in up to 25.3% of cases. This may relate to the anatomical location of the procedure, posing it at increased risk of wound tension, potentially increasing the risk of dehiscence. The risk of postoperative complications in panniculectomy was primarily driven by BMI at the time of surgery and diabetes. An analysis of the American College of Surgeons National Quality Improvement Program database demonstrated that diabetes is an indepedent risk factor for wound dehiscence.^17^ Higher BMI has been extensively described as a risk factor for postoperative complications in surgical literature, including in the context of body contouring.^18,19^

Smoking is also a well-established risk factor for wound complications with body-contouring procedures; existing literature has found that there are significantly higher rates of wound complications and dehiscence among active smokers undergoing body-contouring procedures, including abdominoplasty, compared with nonsmokers (28121897, 12711974). In this study cohort, ∼5% of patients reported smoking during the consultation period. However, per institutional policy, all patients were required to confirm smoking cessation for a minimum of 4 weeks prior to surgery, resulting in zero active smokers at the time of elective body-contouring surgery. Although recent smoking cessation does not fully normalize the risk compared with never smokers, studies have shown that cessation for at least 4 weeks is associated with significant reductions in pulmonary complications and improved wound-healing outcomes (22187226, 40840082). Therefore, a significantly increased risk for postoperative complications was not expected. Accordingly, these findings reflect an institutional approach emphasizing strict patient selection.

Additionally, skin redundancy and altered interstitial compartments can create susceptibility for fluid accumulation in the postoperative setting. Impaired tissue quality in combination with protein, albumin, and micronutrient deficiencies often associated with patients that have experienced MWL can delay wound healing and increase infection risk when compared with patients of normal BMI.^20-22^

Despite finding no difference in complications by weight-loss method, patients with a history of bariatric surgery and GLP-1 RA pharmacotherapy use achieved the greatest percent total weight loss in this cohort, suggesting that combination therapy may represent an effective strategy for patients who plateau or regain weight following surgery alone. Although this finding should be interpreted in the context of selection bias, as the cohort is limited to patients who ultimately pursued body-contouring surgery, similar trends have been reported in obesity management literature (doi: 10.1001/jama.2025.20301, 38488042, 38502519). Because 10% to 30% of bariatric patients experience inadequate weight loss or regain some weight, this study may be informative for bariatric surgeons when counseling patients on adjunctive therapies after surgical weight loss (doi: 10.1001/jama.2025.20301). For plastic surgeons, awareness that patients presenting after combination therapy may have experienced more profound weight loss is clinically relevant. Patients with a history of combination therapy may require more perioperative nutritional supplementation, as vitamin and micronutrient levels, bone health, and muscle mass may be more difficult for these patients to maintain (doi: 10.1001/jama.2025.20301).

This study provides surgeons with important information pertaining to their care of patients who have experienced MWL through differing modalities. Bariatric surgery and injectable GLP-1 RA−based medications have gained popularity as the prevalence of obesity continues to rise in the United States. With the increased utilization of different weight-loss modalities, there is a greater demand for surgical expertise in body contouring to help with the cosmetic and aesthetic challenges accompanying changes in body composition after weight loss. In this cohort, no specific weight-loss modality was associated with increased postoperative complications, indicating that weight-loss modality should not be used as a standalone risk stratifier. Instead, given the observed associations with postoperative risk and factors such as BMI at the time of surgery, diabetes mellitus, and hypertension, along with literature demonstrating that weight-loss patients may require more nutritional support, patients should undergo comprehensive metabolic and medical optimization, with particular attention to potential cumulative risk when multiple comorbidities coexist, to limit possible complications with body-contouring surgeries.

The findings of this study should be interpreted in the context of its methodological limitations. First, the retrospective and single-center methodological design limits the generalizability to other practices. Second, detailed information on the duration, dosage, and adherence to injectable GLP-1 RA pharmacotherapy was not available, limiting the assessment of how the medication might influence postoperative outcomes. Similarly, factors such as nutrition status (eg, albumin and micronutrient status), perioperative optimization protocols, and surgeon variability were not captured and may contribute to residual confounding. Although smoking history was recorded, limited data on cumulative exposure, total cessation duration, and postoperative smoking resumption constrained interpretation of smoking-related risk. Third, the distribution of patients across weight-loss modalities was imbalanced, with the surgical group comprising the majority of the cohort. Although no statistically significant differences were observed between modalities, the smaller sample sizes in the injectable GLP-1 RA pharmacotherapy, combination, and lifestyle groups may have limited the ability to detect clinically relevant differences because of limited power. The finding of no difference between weight-loss modality and postoperative complication implies a lack of association rather than equivalence of modalities. Despite the above-mentioned limitations, this study represents one of the largest analyses to date, exploring the weight-loss patterns and postoperative complication rates in patients who underwent body-contouring surgery following weight loss.

Future research in the field should include prospective, multicenter studies with detailed information on pharmacotherapy data. This will enhance the understanding of how these newer generation injectable GLP-1 RA pharmacological agents may influence outcomes in body-contouring surgery. Furthermore, investigations of the nutritional status, body composition, and tissue quality in injectable GLP-1 RA patients may provide insight into the metabolic and anatomical changes that these patients exhibit.

## CONCLUSIONS

This single-institution, comparative study demonstrates that weight-loss modality was not associated with postoperative complications in patients undergoing body-contouring surgery. However, because the injectable GLP-1 RA pharmacotherapy and combination therapy cohorts were small, the study may be underpowered to detect clinically meaningful differences and therefore cannot establish equivalence among nonsurgical weight-loss strategies. Instead, BMI at the time of surgery, diabetes mellitus, and hypertension were the primary independent predictors of postoperative complications, including seroma, highlighting the importance of comorbidity optimization in perioperative risk stratification.

## Supplemental Material

This article contains supplemental material located online at https://doi.org/10.1093/asj/sjag049.

## Supplementary Material

sjag049_Supplementary_Data
