# Supplementary material for: Post Weight Loss Body Contouring Surgery: Complication Rates Following Bariatric Surgery, Injectable Glucagon-like Peptide-1 Receptor Agonist Pharmacotherapy, Combination Therapy, and Lifestyle Modification
Source: Aesthet Surg J. 2026 Feb 25;46(9):1029–36. doi: 10.1093/asj/sjag049 (PMC13481096; doi:10.1093/asj/sjag049)
Supplement: Supplementary file 1 [file sjag049_supplementary_data.docx]

**Supplemental Table 1**. Baseline Characteristics Of Brachioplasty Patients With and Without Complications

|  | No  (n=96) | Yes  (n=13) | p-value |
| --- | --- | --- | --- |
| Sex at Birth (n, %)  Female  Male | 86 (89.6%) 10 (10.4%) | 13 (100%) 0 (0%) | 0.605 |
| Age at body contouring surgery (years, mean (SD)) | 48.0 (11.4) | 46.1 (7.02) | 0.561 |
| Massive weight loss (MWL) (n, %)  No  Yes | 4 (4.2%) 92 (95.8%) | 0 (0%) 13 (100%) | 1 |
| Maximum BMI (kg/m2, mean (SD)) | 53.8 (10.1) | 51.5 (10.8) | 0.444 |
| BMI at body contouring surgery (n, %) | 31.1 (6.79) | 29.1 (3.50) | 0.300 |
| Percent change in weight (%, mean (SD)) | 41.6 (10.7) | 42.4 (7.84) | 0.785 |
| Method (n, %))  Surgical  Combination  Lifestyle  Medication | 76 (79.2%)  73 (9.9%)  6 (6.3%)  0 (0%) | 108 (69.2%)  17 (10.9%)  21 (13.5%)  10 (6.4%) | 0.723 |
| Smoker (n, %)  No  Yes | 93 (96.9%) 3 (3.1%) | 13 (100%) 0 (0%) | 1 |
| Diabetes (n, %)  No  Yes | 76 (79.2%) 20 (20.8%) | 11 (84.6%) 2 (15.4%) | 1 |
| Hypertension (n, %)  No  Yes | 54 (56.3%) 41 (42.7%) | 8 (61.5%) 5 (38.5%) | 0.982 |
| Iron deficient anemia (n, %)  No  Yes | 74 (77.1%) 22 (22.9%) | 10 (76.9%) 3 (23.1%) | 1 |
| Concurrent Body Contouring (n, %)  No  Yes | 21 (21.9%) 75 (78.1%) | 3 (23.1%) 10 (76.9%) | 1 |
| Incision type (n, %)  Liposuction and avulsion  Standard excision | 45 (46.9%)  51 (53.1%) | 4 (30.8%)  9 (69.2%) | 0.425 |

**Supplemental Table 2**. Baseline Characteristics of Thighplasty Patients With and Without complications

|  | No  (n=53) | Yes  (n=30) | p-value |
| --- | --- | --- | --- |
| Sex at Birth (n, %)  Female  Male | 45 (84.9%) 8 (15.1%) | 26 (86.7%) 4 (13.3%) | 1 |
| Age at body contouring surgery (years, mean (SD)) | 48.1 (12.3) | 50.1 (10.3) | 0.440 |
| Massive weight loss (MWL) (n, %)  No  Yes | 4 (7.5%) 49 (92.5%) | 0 (0%)  30 (100%) | 0.291 |
| Maximum BMI (kg/m2, mean (SD)) | 55.4 (12.5) | 61.3 (17.8) | 0.077 |
| BMI at body contouring surgery (n, %) | 31.3 (7.09) | 37.1 (12.7) | <0.001 |
| Percent change in weight (%, mean (SD)) | 42.5 (10.3) | 38.6 (12.0) | 0.121 |
| Method (n, %))  Surgical  Combination  Lifestyle  Medication | 37 (69.8%)  7 (13.2%)  6 (11.3%)  3 (5.7%) | 26 (86.7%)  1 (3.3%)  3 (10.0%)  0 (0%) | 0.257 |
| Smoker (n, %)  No  Yes | 52 (98.1%)  1 (1.9%) | 29 (96.7%)  1 (3.3%) | 1 |
| Diabetes (n, %)  No  Yes | 40 (75.5%)  12 (22.6%) | 20 (66.7%)  10 (33.3%) | 0.453 |
| Hypertension (n, %)  No  Yes | 28 (52.8%)  25 (47.2%) | 13 (43.3%)  17 (56.7%) | 0.547 |
| Iron deficient anemia (n, %)  No  Yes | 37 (69.8%)  16 (30.2%) | 21 (70.0%)  9 (30.0%) | 1 |
| Concurrent Body Contouring (n, %)  No  Yes | 29 (54.7%) 24 (45.3%) | 18 (60.0%) 12 (40.0%) | 0.813 |
| Incision type (n, %)  Vertical  Horizontal/Transverse  Combined (T or L shape) | 37 (69.8%)  10 (18.9%)  6 (11.3%) | 20 (66.7%)  4 (13.3%)  6 (20.0%) | 0.538 |

**Supplemental Table 3**. Baseline Characteristics of Breast Patients With and Without Complications

|  | No  (n=146) | Yes  (n=33) | p-value |
| --- | --- | --- | --- |
| Sex at Birth (n, %)  Female  Male | 143 (97.9%) 3 (2.1%) | 33 (100%) 0 (0%) | 1 |
| Age at body contouring surgery (years, mean (SD)) | 46.4 (10.5) | 46.0 (10.5) | 0.846 |
| Massive weight loss (MWL) (n, %)  No  Yes | 10 (6.8%) 136 (93.2%) | 1 (3.0%)  32 (97.0%) | 0.692 |
| Maximum BMI (kg/m2, mean (SD)) | 48.8 (9.04) | 50.3 (10.1) | 0.422 |
| BMI at body contouring surgery (n, %) | 30.1 (4.89) | 30.6 (6.57) | 0.642 |
| Percent change in weight (%, mean (SD)) | 37.2 (10.6) | 38.7 (8.36) | 0.447 |
| Smoker (n, %)  No  Yes | 144 (98.6%) 2 (1.4%) | 33 (100%) 0 (0%) | 1 |
| Diabetes (n, %)  No  Yes | 119 (81.5%) 27 (18.5%) | 25 (75.8%) 8 (24.2%) | 0.611 |
| Hypertension (n, %)  No  Yes | 89 (61.0%) 57 (39.0%) | 23 (69.7%) 10 (30.3%) | 0.461 |
| Iron deficient anemia (n, %)  No  Yes | 115 (78.8%) 31 (21.2%) | 27 (81.8%) 6 (18.2%) | 0.878 |
| Concurrent Body Contouring (n, %)  No  Yes | 16 (11.0%) 130 (89.0%) | 7 (21.2%) 26 (78.8%) | 0.146 |
| Concurrent Breast/ Axillary Liposuction (n, %) No  Yes | 1 (0.7%) 145 (99.3%) | 0 (0%) 33 (100%) | 1 |
| Breast Surgery (n, %)  Reduction  Mastopexy  Augmentation/Augpexy | 39 (26.7%)  75 (51.4%)  28 (19.2%) | 14 (42.4%)  16 (48.5%)  3 (9.1%) | 0.151 |
| Implant present (n, %))  No  Yes | 114 (78.1%) 28 (19.2%) | 30 (90.9%) 3 (9.1%) | 0.235 |
